# A 12-Lead ECG-Based System With Physiological Parameters and Machine Learning to Identify Right Ventricular Hypertrophy in Young Adults

**DOI:** 10.1109/JTEHM.2020.2996370

**Published:** 2020-05-21

**Authors:** Gen-Min Lin, Henry Horng-Shing Lu

**Affiliations:** 1 Department of Preventive MedicineFeinberg School of MedicineNorthwestern UniversityChicagoIL60611USA; 2 Department of MedicineHualien Armed Forces General Hospital 63426 Hualien97144Taiwan; 3 Department of MedicineTri-Service General Hospital, National Defense Medical CenterTaipei11490Taiwan; 4 Institute of Statistics, National Chiao Tung University 34914 Hsinchu30010Taiwan

**Keywords:** Electrocardiographic system, right ventricular hypertrophy, support vector machine, physiological parameters, young adults

## Abstract

Objective: The presence of right ventricular hypertrophy (RVH) accounts for approximately 5-10% in young adults. The sensitivity estimated by commonly used 12-lead electrocardiographic (ECG) criteria for identifying the presence of RVH is under 20% in the general population. The aim of this study is to develop a 12-lead ECG system with the related information of age, body height and body weight via machine learning to increase the sensitivity and the precision for detecting RVH. Method: In a sample of 1,701 males, aged 17–45 years, support vector machine is used for the training of 31 parameters including age, body height and body weight in addition to 28 ECG data such as axes, intervals and wave voltages as the inputs to link the output RVH. The RVH is defined on the echocardiographic finding for young males as right ventricular anterior wall thickness > 5.5 mm. Results: On the system goal for increasing sensitivity, the specificity is controlled around 70-75% and all data tested in the proposed method show competent sensitivity up to 70.3%. The values of area under curve of receiver operating characteristic curve and precision-recall curve using the proposed method are 0.780 and 0.285, respectively, which are better than 0.518 and 0.112 using the Sokolow-Lyon voltage criterion, respectively, for detecting unspecific RVH. Conclusion: We present a method using simple physiological parameters with ECG data to effectively identify more than 70% of the RVH among young adults. Clinical Impact: This system provides a fast, precise and feasible diagnosis tool to screen RVH.

## Graphical abstract

This paper uses a machine learning method to train physiological parameters and ECG features to detect Right Ventricular Hypertrophy (RVH). We developed a clinically effective ECG system with simple physiological parameters to screen for RVH in a large sample of young adults. Compared with traditional ECG criteria, the proposed technique provides superior performances with regard to sensitivity, precision, F1 score, and the AUCs of ROC curves and PR curves.

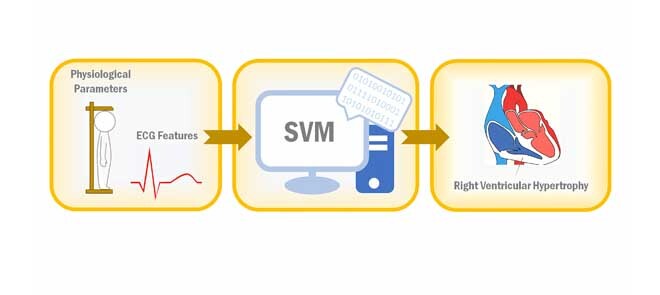

## I. Introduction

The applications of artificial intelligence (AI) have emerged in many aspects worldwide based on the huge improvements in technology and big data availability. Machine learning is a technique, integrating AI and computation, back and forth to find the best outcome in the model which has been successfully used in the decision making for clinical disease diagnosis and the risk prediction [1]–[13]. For example, [5] uses the machine learning by training hundreds of electrocardiographic (ECG) features to identify the pathological hypertrophic cardiomyopathy (HCM) [14]. Relying on the machine learning or deep learning techniques, the physicians in next generation can make more accurate and fast judgements on the prognosis and disposals of a disease. In addition, AI can provide relevant and cost-effective medical service in the medically underserved regions.

Right ventricular hypertrophy (RVH) is mostly secondary to some pathological conditions such as chronic lung disease [15], pulmonary embolism [16], systemic arterial hypertension with left ventricular hypertrophy [17], [18] and primary pulmonary arterial hypertension [19], [20]. In some cases, RVH is involved in congenital cardiac diseases such as atrial or ventricular septal defects [21], pulmonary valve stenosis [22] and hypertrophic cardiomyopathy (HCM) [23]. The presence of RVH has been associated with heart failure and cardiovascular disease events in middle and old-aged individuals [24], [25]. Since most of the RVH phenotypes represent a presence of underlying pathologic diseases, it is important to identify it at younger ages. However, the RVH prevalence in young adults is low, approximately 5-10% [26], making it difficult to be screened out. The currently most used tool for detecting the presence of RVH among the general population is 12-lead surface electrocardiography (ECG) [27]. Several ECG-based criteria such as the interpretations by Myers *et al.* and Sokolow-Lyon have been proposed for years [28], [29]; however, the performances of these ECG-based criteria for RVH consistently yield high specificity but low sensitivity. To our best knowledge, there have been a few studies implemented by machine learning and deep learning for the ECG features to detect left ventricular hypertrophy [4], whereas the performance is only suboptimal in the general population. It is possible that a use of machine learning by solely the ECG features might not be good enough to fit for clinical requirements. In another respect, Tison *et al.* use the deep learning of convolutional neural network for training the ECG features to predict pulmonary arterial hypertension, which shows an excellent result [6]. However, the machine learning methods have rarely been utilized for detecting the presence of RVH.

In this paper, we use a large sample of the military members taking age, body height and body weight as well as a number of ECG features into considerations for machine learning by the support vector machine (SVM) technique to relate to RVH. The rest of this paper is organized as follows. The materials and pre-test results for input features are revealed in Section II. Section III presents the proposed algorithm regarding the system for screening out RVH in detail. The experimental results are displayed in Section IV. We conclude this paper in Section V.

## II. Data Collection and Features Selection

### A. Data Collection

This study includes a sample of 1,701 military males of 17–45 years from the ancillary cardiorespiratory fitness and hospitalization events in armed forces (CHIEF) substudy implemented in the Hualien Armed Forces General Hospital in Hualien city, Taiwan, R.O.C. Each participant underwent a 12-lead ECG and a transthoracic echocardiography at the same visit for an annual routine health examination. The design and rationale of this study has been described previously [30]–[42]. The 12-lead ECG features were obtained from two ECG manufacturers’ products including CARDIOVIT MS-2015 (Schiller AG, Baar, Switzerland) and TC70 CARDIOGRAPH (Philips, Amsterdam, Netherlands). The ECG signal in each lead was recorded with a duration of 2.5 seconds and the sampling frequency of 500 Hz. The echocardiography was operated by utilizing the IE33 (Philips, Amsterdam, Netherlands). All the ECG and echocardiography procedures were performed by a senior certificated technician. The 28 ECG features used in the proposed method include heart rate, the axes of P, QRS, and T waves in Lead II, and the durations of P wave, PR interval, QRS interval, QT interval and QTc interval in Lead II, and the amplitudes of R waves in limb Leads I, II, III, aVR, aVL and aVF and S wave in Lead aVL, and the amplitudes of R and S waves in chest Leads V1-V6, which are obtained by either CARDIOVIT MS-2015 or TC70 CARDIOGRAPH. In addition, a population of 176 military females of ages 17–42 years from the ancillary CHIEF substudy, is treated as another test set using the male model trained by the SVM machine learning for age, anthropometrics and ECG features. The comparison methods are the Sokolow-Lyon voltage criterion for RVH [29], defined as a composite of amplitudes R-V1+(S-V5 or S-V6) > 10.5 mm for both males and females, and Myers *et al.* [28] voltage criterion, defined as (R-V1/S-V1 ratio > 1) or (R-V5/S-V5 ratio or R-V6/S-V6 ratio < 1) or (R-V1 > 6 mm) for both males and females, respectively, where the voltage of 0.1 mV represents 1 mm.

The diagnosis of RVH is based on the recommendations of the American Society of Echocardiography [43]. Quantification of right ventricular wall thickness (RVWT) is measured by M-mode and 2-dimensional methods at the onset of QRS complex of end diastole in echocardiographic parasternal long axis view. Echocardiographic RVH for young male adults is defined to be RVWT > 5.5 mm which is approximately the 95^th^ percentile in the military males [44]. In addition, echocardiographic RVH for young female adults is defined to be RVWT > 5.2 mm, which is determined based on the 95^th^ percentile of our military females. The cut-off points for the echocardiographic RVH for both male and female adults are suggested as RVWT > 5.0 mm which are fit for the suggestion by the American Society of Echocardiography [43]. To devise the proposed machine learning method, the data for the male samples are partitioned into 80% for training with cross validation and 20% for test. This study protocol has been approved by the Institutional Review Broad of Mennonite Christian Hospital (No. 16-05-008) in Hualien, Taiwan.

### B. Pre-Test for Input Features

Several physiological parameters are stepwise added on the 28 ECG features, as the input parameters for SVM machine learning to find the most efficient system for clinical use at the initial stage. These physiological parameters include age, body height, body weight, waist circumference, systolic blood pressure (SBP) and diastolic blood pressure (DBP). The preliminary results of additional physiological parameters and adopted 28 ECG features are listed in Table 1. In the stepwise pre-test, we only use training set and test set for the SVM model to compare the performances of different ECG-based combinations. As revealed from the combinations in Table 1, the largest area under curve (AUC) of Precision-Recall (PR) curve and the competent AUC of Receiver Operating Characteristic (ROC) curve in the test set are observed when age, body height, body weight and the 28 ECG parameters are the inputs to relate to the output RVH. Thus, these 31 parameters are decided as the input features of our machine learning model. The baseline values of each parameter for the study participants are demonstrated in Table 2. The label of RVH is by the criterion of RVWT > 5.5 mm for young males. As revealed in Table 2, the characteristics in those with and those without RVH are presented as mean ± standard deviation for continuous data and compared by independent t-test. A p-value < 0.05 is regarded significant. It is notable that older age, greater body height and body weight are observed in those with echocardiographic RVH.

**TABLE 1:** Preliminary Performances of Additional Physiological Parameters and Adopted 28 ECG Features

ECG (28)	✔	✔	✔	✔	✔
Age	–	✔	✔	✔	✔
Height and Weight	–	–	✔	✔	✔
Waist Circumference	–	–	–	✔	✔
SBP and DBP	–	–	–	–	✔
Number of Input Features	28	29	31	32	34
ROC AUC	0.725	0.758	0.790	0.789	0.800
PR AUC	0.300	0.307	0.361	0.349	0.340

**TABLE 2:** Characteristics of Study Participants (Males)

Features	Total N=1701	Non-RVH N=1526	RVH N=175	p-value
Age (years)	25.30±6.82	24.93±6.61	28.51±7.74	<0.001
Height (cm)	172.03±5.96	171.85±5.97	173.59±5.76	<0.001
Weight (kg)	72.45±12.25	71.41±11.80	81.51±12.40	<0.001
Heart rate (bpm)	66.89±11.90	67.08±11.91	65.18±11.81	0.046
P-II(ms)	106.21±15.24	105.97±15.25	108.28±15.11	0.058
PR-II(ms)	157.30±20.70	156.84±20.67	161.27±20.57	0.007
QRS-II(ms)	97.66±10.49	97.49±10.60	99.12±9.39	0.033
QT-II(ms)	370.57±28.24	369.74±28.20	377.75±27.74	<0.001
QTc-II(ms)	389.40±24.76	389.09±24.88	392.10±23.61	0.129
P axis-II(degree)	44.84±26.94	45.21±26.99	41.66±26.38	0.098
QRS axis-II(degree)	64.95±32.58	66.34±32.00	52.85±35.09	<0.001
T axis-II(degree)	35.77±21.03	36.64±20.71	28.17±22.25	<0.001
R-I(mm)	5.82±2.98	5.61±2.82	7.63±3.64	<0.001
R-II(mm)	13.03±5.01	13.16±5.04	11.88±4.66	0.001
R-III(mm)	8.64±5.94	8.90±5.97	6.41±5.18	<0.001
R-aVR(mm)	1.11±1.24	1.12±1.25	1.04±1.12	0.426
R-aVL(mm)	2.35±2.19	2.20±2.01	3.68±3.11	<0.001
S-aVL(mm)	2.63±3.08	2.67±3.12	2.30±2.64	0.092
R-aVF(mm)	10.68±5.43	10.89±5.45	8.84±4.89	<0.001
R-V1(mm)	3.63±2.25	3.67±2.28	3.32±1.94	0.027
S-V1(mm)	10.37±5.34	10.46±5.33	9.62±5.37	0.048
R-V2(mm)	8.87±4.27	8.83±4.26	9.19±4.32	0.284
S-V2(mm)	16.32±6.90	16.41±6.84	15.53±7.30	0.110
R-V3(mm)	13.55±6.30	13.59±6.40	13.24±5.41	0.427
S-V3(mm)	8.64±5.35	8.56±5.31	9.39±5.58	0.049
R-V4(mm)	20.05±7.03	20.19±7.14	18.85±5.90	0.006
S-V4(mm)	5.48±4.19	5.39±4.15	6.33±4.46	0.005
R-V5(mm)	20.12±5.91	20.09±5.96	20.35±5.41	0.572
S-V5(mm)	3.44±3.01	3.37±2.95	4.03±3.46	0.017
R-V6(mm)	16.59±5.06	16.45±5.07	17.76±4.99	0.001
S-V6(mm)	2.04±2.03	2.01±1.99	2.33±2.39	0.088

## III. Proposed Method

We use the 31 input parameters consisting of age, body height, body weight and the 28 ECG features for machine learning on the basis of the preliminary results from the pre-test. The SVM model for these features to relate to the presence of RVH in the young military males is chosen as machine learning technique. The reasons for selecting the SVM model are according to its merits of memory efficiency, effectiveness in high dimensional spaces and very successful discriminative models in many applications [3], [5], [12], [13], [45]. In addition, SVM could provide efficient operation process by taking less training time and running time. Therefore, the SVM is utilized as the machine learning technique which can be practical in an ECG equipment.

### A. Data Pre-Processing and Cross Validation

Because of different dynamic ranges for various input features, the Min-Max normalization is used to normalize the original data of 31 input features into the interval [0-1]. A linear transformation on the original data for each feature is performed by Min-Max normalization for data pre-processing.

**FIGURE 1.**
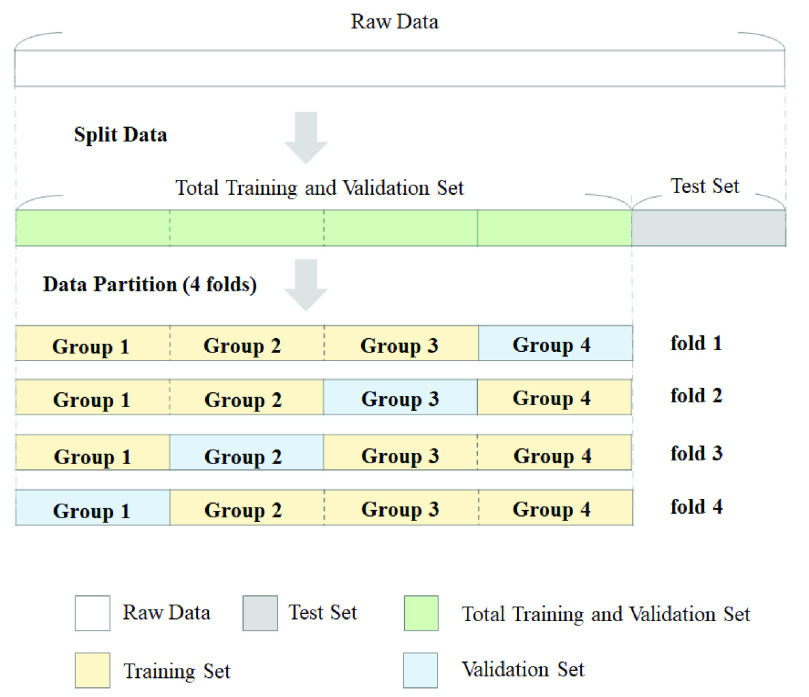
Data partition of the datasets and cross validation.

The partition of experimental data is exhibited in Fig. 1. The normalized data of 1,701 military males are divided into the total training and validation set and the test set with 4:1 ratio. The total training and validation set is segmented into four equal sample size groups. Within the four groups, one group is taken as the validation set for validating the model, and the other three groups are used as the training set. Fig. 1 also shows the data partition of four folds. Each fold has similar proportions of Non-RVH and RVH cases. The 4-fold cross validation process repeats the training and validation procedures for four times. Each of the four groups is utilized once as the validation set. The values of the area under curve (AUC) of the PR curves for the four folds are averaged and taken as a single performance.

Table 3 lists the data numbers composed by four folds. Non-RVH samples predominate and RVH samples occupy only a small percentage in our data since the prevalence of RVH in the young adults is about 10%. For example, in the 1st cross validation, the numbers for training data and validation data are 1,020 (Non-RVH: 921, RVH: 99) and 340 (Non-RVH: 300, RVH: 40), respectively. This imbalance phenomenon between Non-RVH and RVH groups is evident. In [46], Chawla *et al.* propose the synthetic minority over-sampling technique (SMOTE), which is a popular over-sampling method. We apply SMOTE to solve the problem of imbalance data. SMOTE mainly creates new minority class samples by selecting a near minority class neighbor randomly and interpolating. In the viewpoint of geometry, the process of SMOTE can be regarded as the interpolation between two minority class samples and thus expand the decision space for RVH samples. It benefits the SVM classifier to provide a better prediction rate on RVH samples.

**TABLE 3:** Data Numbers in the Training and Validation Set for 4-Fold Cross Validation

Fold	Data	Non-RVH	RVH	Total
1st	Training Set	921	99	1020
	Pre-processed by SMOTE	921	921	1842
	Validation Set	300	40	340
2nd	Training Set	922	98	1020
	Pre-processed by SMOTE	922	922	1844
	Validation Set	299	41	340
3rd	Training Set	909	111	1020
	Pre-processed by SMOTE	909	909	1818
	Validation Set	312	28	340
4th	Training Set	911	109	1020
	Pre-processed by SMOTE	911	911	1822
	Validation Set	310	30	340

As shown in Table 3, the SMOTE is utilized in the process of 4-fold cross validation. The training data for RVH groups are pre-processed by SMOTE to be the same amount with the numbers of non-RVH groups as 921, 922, 909 and 911, respectively, for the four folds.

### B. Machine Learning Model

The binary classifier, support vector machine [47]–[49], is used by the proposed method for machine learning. SVM estimates the hyperplane that best discriminates Non-RVH and RVH classes in a high dimensional space according to a maximum separation margin criterion. Generally speaking, a good separation is realized by the hyperplane that has the largest distance to the nearest training data points of Non-RVH and RVH classes, since the larger the margin, the lower the generalization error of the SVM classifier. Soft-margin SVM, which is adopted in our method, allows a certain number of mistakes and preserves margin as wide as possible and some outliers are inside or on the incorrect side of the margin.

A training vector in Non-RVH or RVH class with associated label is processed by Min-Max normalization. We synthesize and increase the minority data (RVH group) in the training set by using SMOTE. The linear SVM classifier generates the weight vector to construct the hyperplane, which is obtained by solving the objective function with the L2 norm regularization and loss function for the soft–margin SVM evaluated on the training set and weighted by hyperparameter *C.* The hyperparameter $C$ decides the trade-off between minimizing the training error and maximizing the margin. To make the decision based on the training data, the output class (Non-RVH or RVH class) of validation set or test set can be predicted by the input feature vector.

**FIGURE 2.**
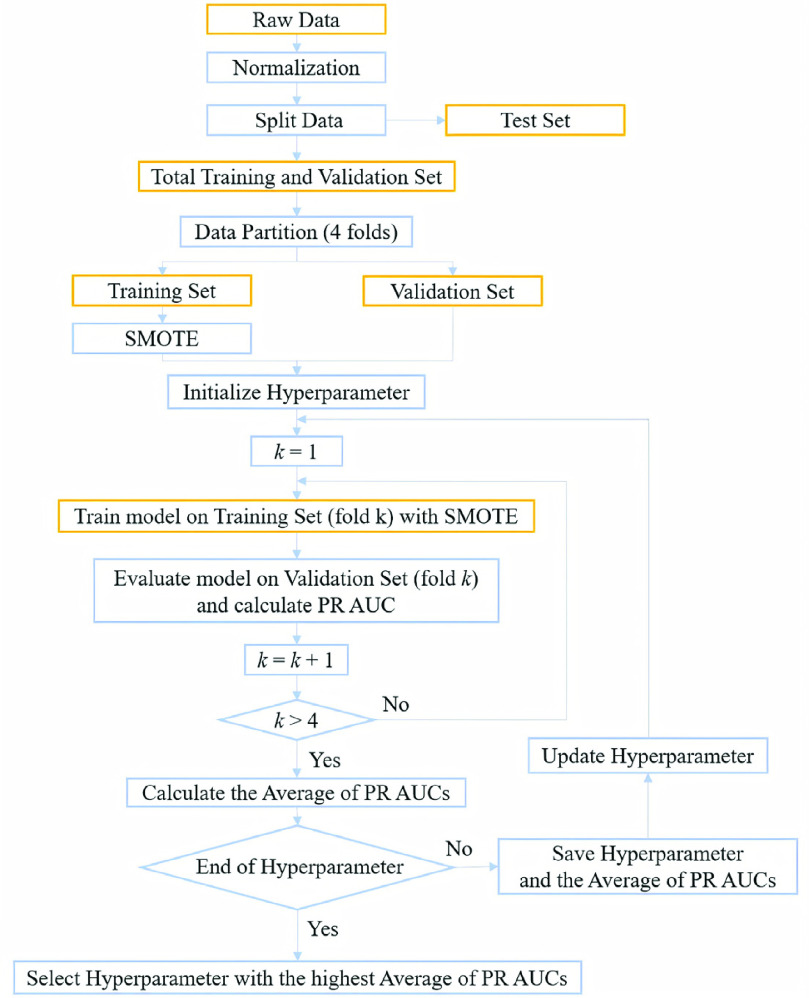
Flowchart for the selection of optimized hyperparameter.

The optimization for the selection of hyperparameter $C$ is implemented by grid search. The training process by grid search is iterated until the hyperparameter reaches to end value. As shown in Fig. 2, the optimized hyperparameter is chosen with the highest average PR AUC of the 4-fold cross validation among the candidates of $C$.

**FIGURE 3.**
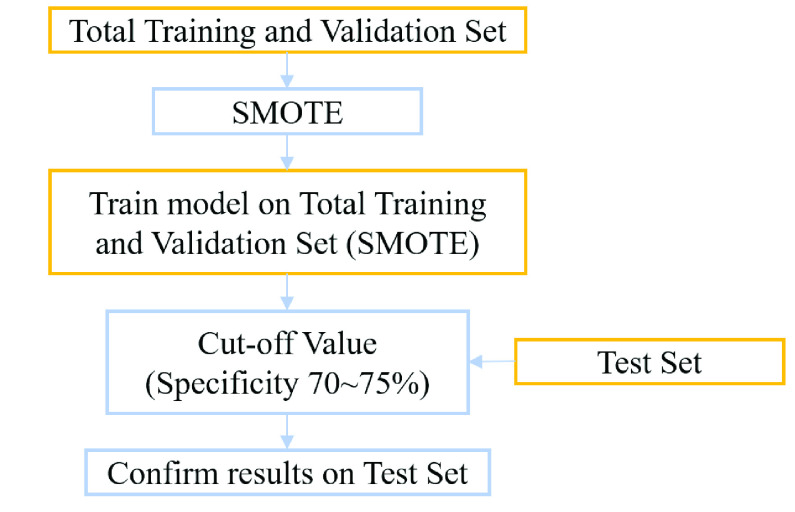
Flowchart of training and test procedures of proposed method.

After the optimized hyperparameter is determined, the SVM model is trained by the data in the total training and validation set as shown in Fig. 3. The data in total training and validation set for RVH group are pre-processed by SMOTE, and the number is raised to 1,221 as shown in Table 4.

**TABLE 4:** Data Numbers of Total Data

Data	Non-RVH	RVH	Total
Total Training and Validation Set	1221	139	1360
Pre-processed by SMOTE	1221	1221	2442
Test Set	305	36	341
Total Data	1526	175	1701

## IV. Experimental Results

The proposed RVH screening method is coded by scikit learn v0.20.2 software with Python [50]. The initial test value, the increment and the end test value of the hyperparameter are listed in Table 5.

**TABLE 5:** Hyperparameter Optimization

Model	Hyperparameter	Initial value	End value	Interval	Optimum value
Support Vector Machine (Linear)	$C$	0.02	1	0.001	0.189
Support Vector Machine (RBF)	$C$ Gamma	10 0.001	20 0.1	0.01 0.001	17.09 0.005
Random Forest	Number of Trees	50	300	1	69
Gradient Boosting Decision Tree	Maximum Tree Depth	1	25	1	7

### A. Performance Measurement

To find the most appropriate test cut-off probability [51] for the SVM method, the specificity around 70-75% is chosen as the criterion as shown in Fig. 3. Performance evaluation consists of several standard measurements including accuracy, specificity, sensitivity (recall), precision, F_1_ score, the AUC of ROC curve and the AUC of PR curve [52], [53].

Accuracy, specificity, sensitivity(recall) and precision are defined by true positive (*TP*), true negative (*TN*), false positive (*FP*) and false negative (*FN*) as listed in (1)–(4). F_1_ score represents the harmonic average of the precision and recall as denoted in (5).

\begin{align*} Accuracy=&\frac {TP+TN}{TP+TN+FP+FN}, \tag{1}\\ Specificity=&\frac {TN}{TN+FP}, \tag{2}\\ Sensitivity\left ({Recall }\right)=&\frac {TP}{TP+FN}, \tag{3}\\ Precision=&\frac {TP}{TP+FP}, \tag{4}\\ F_{1}score=&\frac {2\times Precision\times Recall}{Precision+Recall}.\tag{5}\end{align*}

### B. Results and Discussion

**FIGURE 4.**
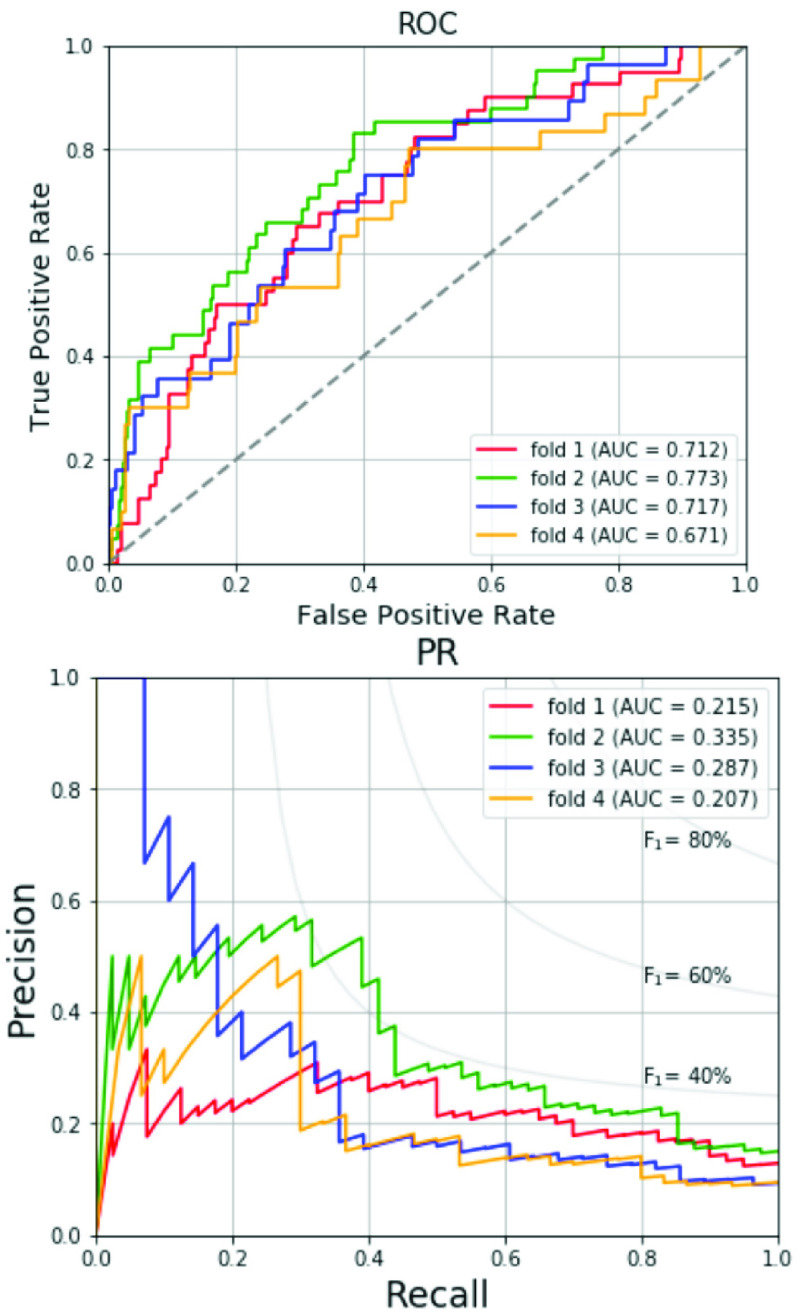
ROC and PR curves for the 4-fold cross validation.

**FIGURE 5.**
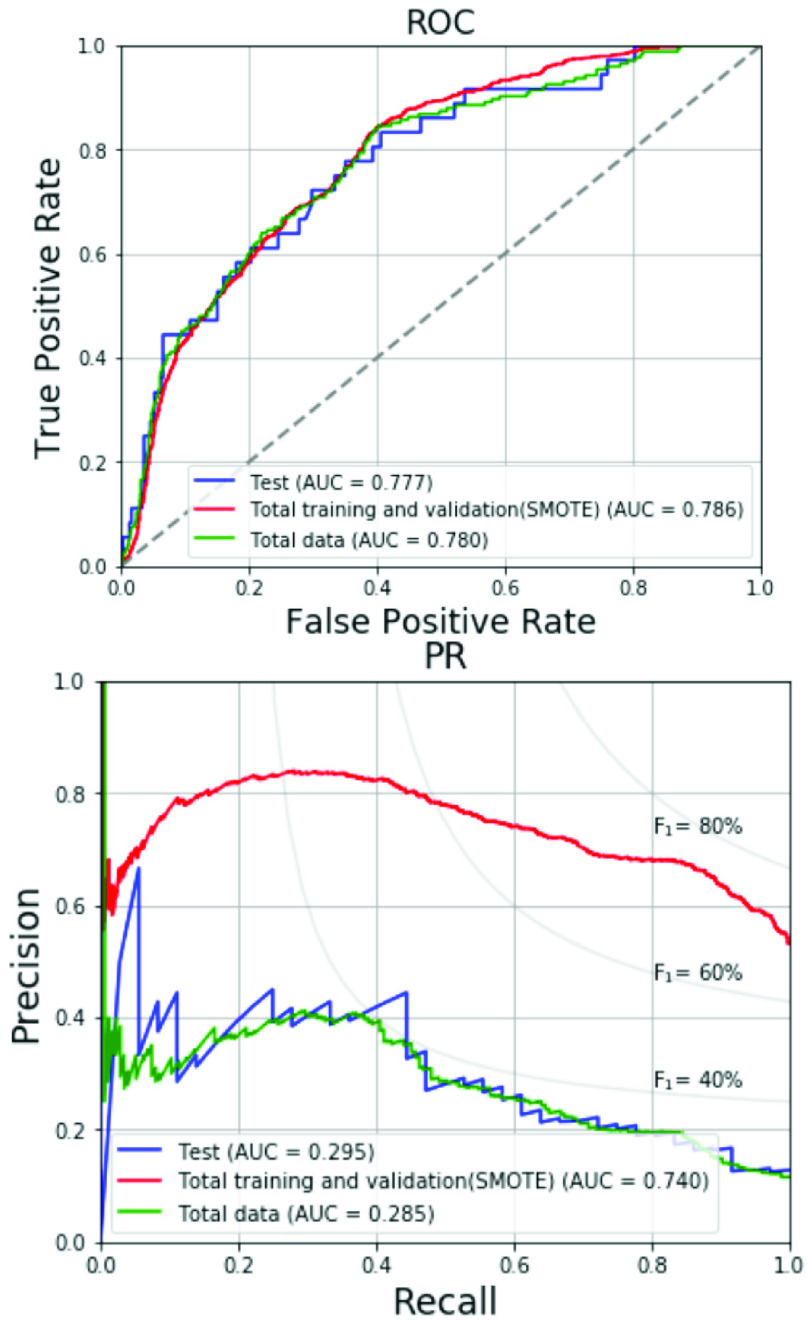
ROC and PR curves of proposed method for various datasets.

**FIGURE 6.**
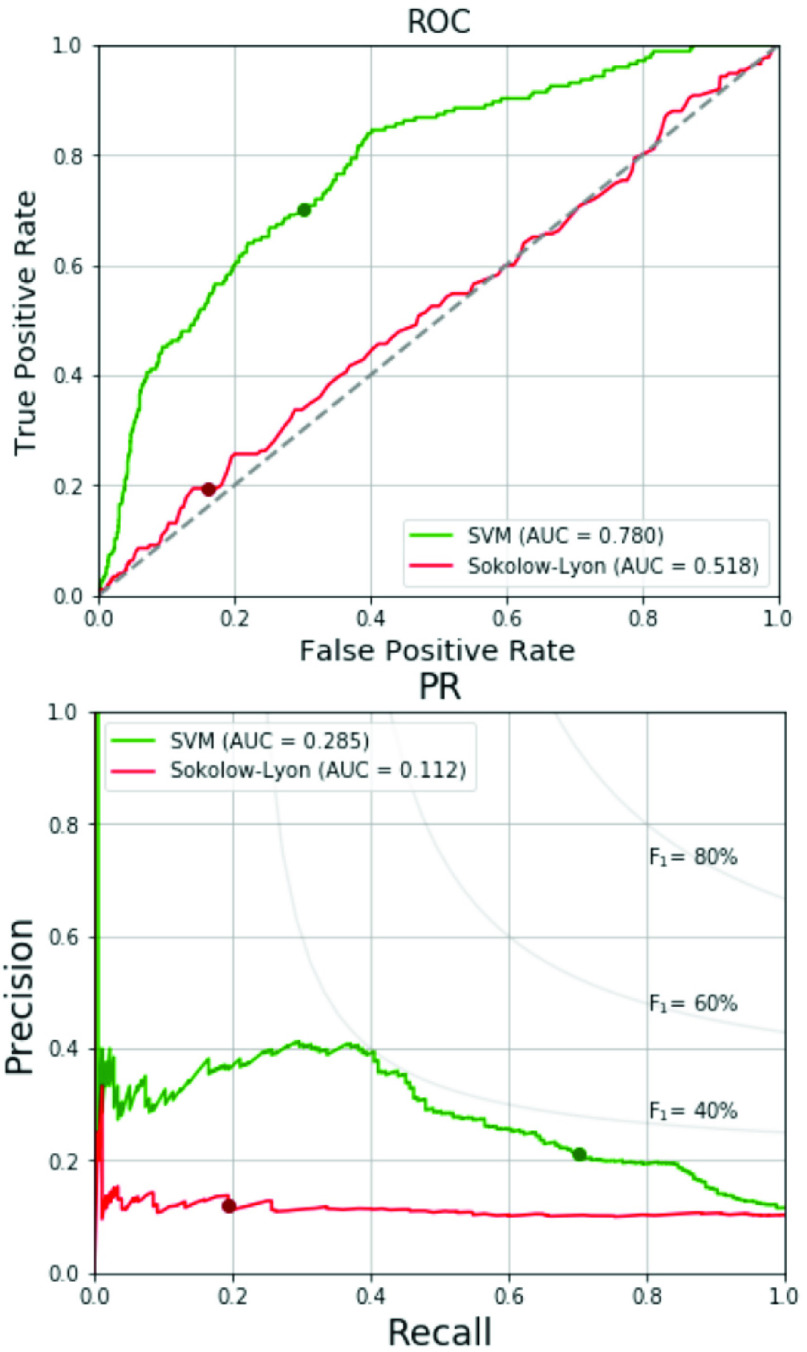
ROC and PR curves of proposed method and traditional ECG voltage criteria.

Table 6 tabulates the data numbers and screening results for RVH of the 4-fold cross validation with the optimized hyperparameter. In the validation sets, the RVH prevalence is ranged from 8.2-12.1% as shown in Table 6. The values of F_1_ score, the AUCs of ROC and PR curves are similar across the four folds. Fig. 4 shows the respective ROC curves and PR curves for the four folds. The average AUC of ROC curve is 0.718 and the average AUC of PR curve is 0.261. The prediction results of the total training and validation set, test set and total data are listed in Table 7. In the total training and validation set, the SMOTE is applied for solving the imbalance in sample sizes between the non-RVH and RVH groups to increase the prevalence of RVH to 50%. Therefore, the precision, the F_1_ score and the AUC of PR curve of the total training and validation set are superior to those of the other two datasets. In the test set and total data, the prevalence of RVH is around 10%. The results of the test set regarding accuracy, specificity, sensitivity, precision and F_1_ score are 70.4%, 70.2%, 72.2%, 22.2% and 34.0%, respectively, which are consistent with the results of the total data. Fig. 5 compares the ROC curves and the PR curves for various datasets including the total training and validation set, the test set and the total data. The three datasets reveal similar AUCs of the ROC curves. We compare the proposed SVM-based machine learning method with the Sokolow-Lyon voltage and the Myers *et al.* voltage criteria for RVH as listed in Table 8. All data of the 1,701 military males are tested. With the specificity of 70.0%, chosen between 70-75%, our SVM-based method provides much better sensitivity 70.3% compared to 19.4% and 15.4% for the Sokolow-Lyon and the Myers *et. al* voltage criteria, respectively. Fig. 6 compares the ROC curves and the PR curves between the Sokolow-Lyon voltage criterion and the proposed SVM-based method for screening RVH. The results show that the proposed SVM-based method has much better performance compared with the traditional Sokolow-Lyon voltage criterion.

**TABLE 6:** Data Numbers and Performances for 4-Fold Cross Validation

	Validation set 1st fold	Validation set 2nd fold	Validation set 3rd fold	Validation set 4th fold	Average
Non-RVH Group	300	299	312	310	–
RVH Group	40	41	28	30	–
Total	340	340	340	340	–
Prevalence Rate	11.8%	12.1%	8.2%	8.8%	–
F_1_-score	30.8%	38.0%	25.0%	25.0%	29.7%
ROC AUC	0.712	0.773	0.717	0.671	0.718
PR AUC	0.215	0.335	0.287	0.207	0.261

**TABLE 7:** Prediction Results of Proposed Method for Various Datasets

	Total training and validation set (SMOTE)	Test set	Total data
Non-RVH Group	1221	305	1526
RVH Group	1221	36	175
Total	2442	341	1701
Prevalence Rate	50.0%	10.6%	10.3%
Accuracy	70.1%	70.4%	70.0%
Specificity	69.9%	70.2%	70.0%
Sensitivity	70.4%	72.2%	70.3%
Precision	70.1%	22.2%	21.2%
F_1_-score	70.2%	34.0%	32.6%
ROC AUC	0.786	0.777	0.780
PR AUC	0.740	0.295	0.285
True Negative	854	214	1068
False Negative	362	10	52
False Positive	367	91	458
True Positive	859	26	123

**TABLE 8:** Performance Comparison of Proposed Method and Traditional ECG Voltage Criteria

	SVM	Sokolow-Lyon	Myers et al.
Cut-off Value	0.495	10.5 mm	–
Accuracy	70.0%	77.3%	74.2%
Specificity	70.0%	83.9%	80.9%
Sensitivity	70.3%	19.4%	15.4%
Precision	21.2%	12.2%	8.5%
F_1_-score	32.6%	15.0%	11.0%
ROC AUC	0.780	0.518	–
PR AUC	0.285	0.112	–
True Negative	1068	1281	1235
False Negative	52	141	148
False Positive	458	245	291
True Positive	123	34	27

We also compare the proposed SVM method using linear kernel with other three machine learning models: SVM with radial basis function (RBF) kernel (non-linear) [54], random forest (RF) [55] and gradient boosting decision tree (GBDT) [56]. The hyperparameter optimization for the three methods is listed in Table 5. The experimental results for test set are shown in Table 9. The performances using SVM models are superior to those of RF and GBDT models. The SVM models with linear and RBF kernels provide similar performances. As described in [57], if the number of input features is large, mapping data to a higher dimensional space may not be needed. In other words, the non-linear mapping may not improve the performance. And only one hyperparameter $C$ is searched for linear kernel instead of (*C, Gamma*) for the RBF kernel.

**TABLE 9:** Performance Comparison of Various Machine Learning Models for Test Set

	SVM (Linear)	SVM (RBF)	RF	GBDT
Accuracy	70.4%	71.0%	69.5%	68.9%
Specificity	70.2%	70.8%	70.5%	70.8%
Sensitivity	72.2%	72.2%	61.1%	52.8%
Precision	22.2%	22.6%	19.6%	17.6%
F_1_-score	34.0%	34.4%	29.7%	26.4%
ROC AUC	0.777	0.780	0.710	0.683
PR AUC	0.295	0.297	0.300	0.330
True Negative	214	216	215	216
False Negative	10	10	14	17
False Positive	91	89	90	89
True Positive	26	26	22	19

**FIGURE 7.**
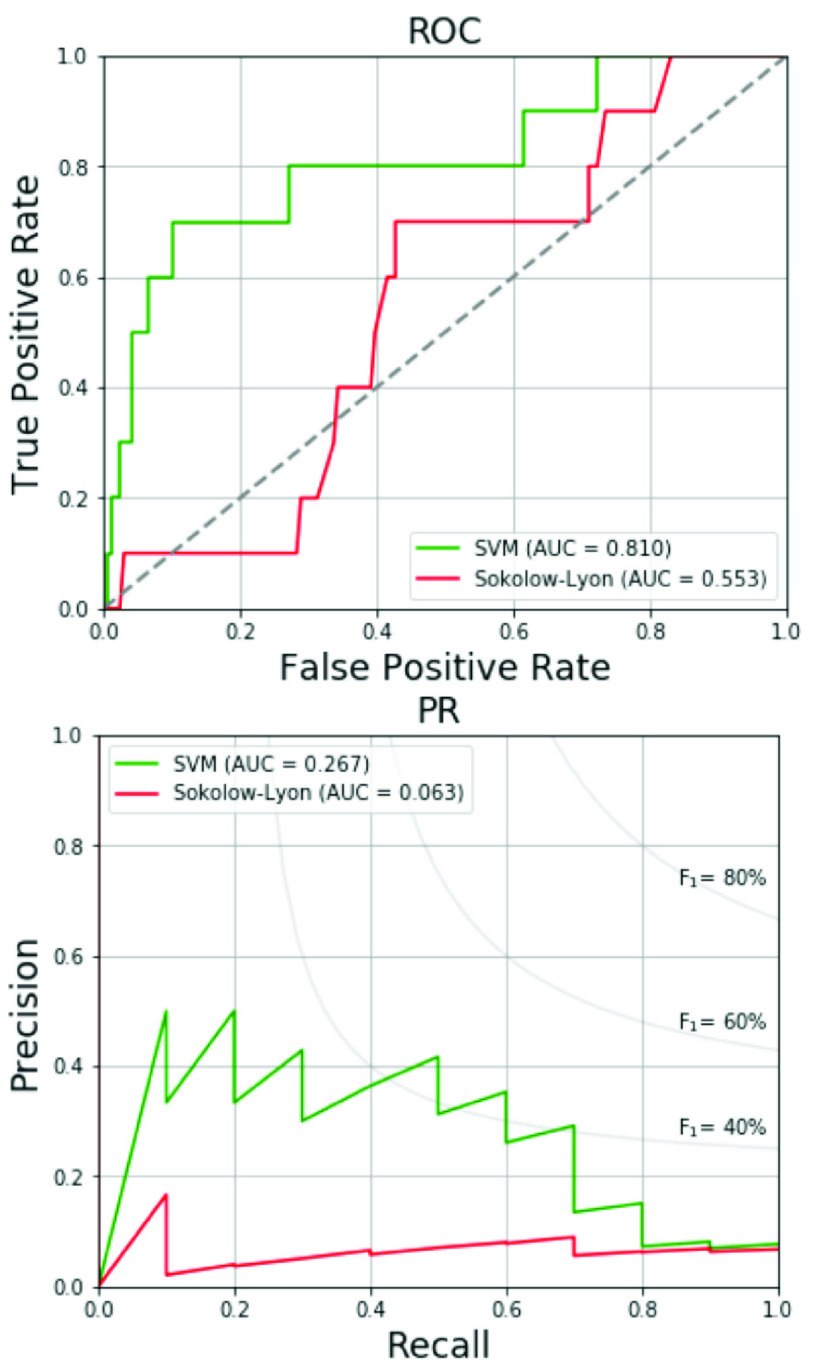
ROC and PR curves of proposed method and traditional ECG voltage criteria for female’s test data.

Furthermore, we also test the CHIEF military female subcohort data (176 military females aged 17–42 years) with the label of echocardiographic RVH by the definition of RVWT > 5.2 mm for young females using the proposed SVM-based model trained by the military young males. The average and standard deviation for each adopted physiological and ECG features of the female participants with and without

echocardiographic RVH are listed in Table 10. Age and body weight are two features with significant differences. The prediction results for the female population are shown in Table 11. The accuracy, specificity, sensitivity, precision and F_1_ score of the female test set using the proposed SVM method with linear kernel are 73.3%, 72.9%, 80.0%, 15.1% and 25.4%, respectively, which are in line with the suboptimal results of the male set. As compared to the SVM method with RBF kernel, the traditional Sokolow-Lyon voltage [29] and Myers *et al.* voltage criteria [28], the proposed SVM method with linear kernel also provides better performance evaluated by F_1_ score, the AUCs of ROC curves and PR curves. Fig. 7 shows the ROC curves and PR curves for the female’s test data.

**TABLE 10:** Characteristics of Study Participants (Females)

Features	Total N=176	Non-RVH N=166	RVH N=10	p-value
Age (years)	25.32±5.35	25.13±5.32	28.50±5.13	0.053
Height (cm)	160.79±4.85	160.71±4.90	162.10±3.77	0.379
Weight (kg)	58.95±9.32	58.18±8.46	71.77±13.54	0.011
Heart rate (bpm)	68.47±11.25	68.38±11.17	69.90±13.09	0.679
P-II(ms)	99.84±15.06	99.58±15.33	104.00±8.99	0.369
PR-II(ms)	147.70±22.74	147.73±23.19	147.20±13.96	0.943
QRS-II(ms)	90.13±12.49	90.26±12.66	88.00±9.38	0.580
QT-II(ms)	383.14±34.91	383.08±35.15	384.00±32.33	0.936
QTc-II(ms)	409.78±29.08	409.56±28.36	413.40±40.99	0.686
P axis-II(degree)	43.57±26.25	44.34±26.01	30.70±28.25	0.111
QRS axis-II(degree)	71.24±32.13	71.77±32.66	62.50±20.83	0.377
T axis-II(degree)	34.49±20.22	33.96±19.98	43.40±23.16	0.152
R-I(mm)	4.14±2.11	4.03±1.98	5.98±3.27	0.093
R-II(mm)	11.30±3.65	11.29±3.68	11.39±3.32	0.936
R-III(mm)	8.35±4.48	8.47±4.53	6.45±2.91	0.167
R-aVR(mm)	0.88±1.16	0.87±1.19	1.10±0.64	0.318
R-aVL(mm)	1.44±1.15	1.40±1.12	2.04±1.45	0.087
S-aVL(mm)	2.75±2.83	2.79±2.86	2.12±2.44	0.472
R-aVF(mm)	9.74±3.85	9.78±3.93	8.99±2.06	0.289
R-V1(mm)	2.71±1.59	2.70±1.58	2.77±1.91	0.902
S-V1(mm)	6.90±3.54	6.84±3.59	7.99±2.41	0.318
R-V2(mm)	6.41±2.94	6.40±2.87	6.45±4.12	0.965
S-V2(mm)	9.68±4.92	9.63±4.93	10.45±4.97	0.612
R-V3(mm)	9.10±4.26	9.11±4.29	9.06±3.89	0.970
S-V3(mm)	5.52±3.81	5.46±3.83	6.53±3.36	0.391
R-V4(mm)	13.16±4.48	13.18±4.50	12.79±4.32	0.791
S-V4(mm)	3.63±2.95	3.60±2.99	4.14±2.10	0.578
R-V5(mm)	13.49±4.11	13.46±4.14	13.97±3.77	0.700
S-V5(mm)	2.49±2.16	2.47±2.21	2.79±1.21	0.654
R-V6(mm)	12.15±3.73	12.08±3.74	13.31±3.47	0.311
S-V6(mm)	1.59±1.58	1.57±1.61	1.92±0.91	0.501

**TABLE 11:** Performance Comparison of Proposed Methods and Traditional ECG Voltage Criteria for Female’s Test Data

	SVM (Linear)	SVM (RBF)	Sokolow-Lyon	Myers et al.
Cut-off Value	0.198	0.500	10.5mm	–
Accuracy	73.3%	74.4%	90.9%	83.5%
Specificity	72.9%	74.7%	95.8%	88.0%
Sensitivity	80.0%	70.0%	10.0%	10.0%
Precision	15.1%	14.3%	12.5%	4.8%
F_1_-score	25.4%	23.7%	11.1%	6.5%
ROC AUC	0.810	0.759	0.553	–
PR AUC	0.267	0.145	0.063	–
True Negative	121	124	159	146
False Negative	2	3	9	9
False Positive	45	42	7	20
True Positive	8	7	1	1

**FIGURE 8.**
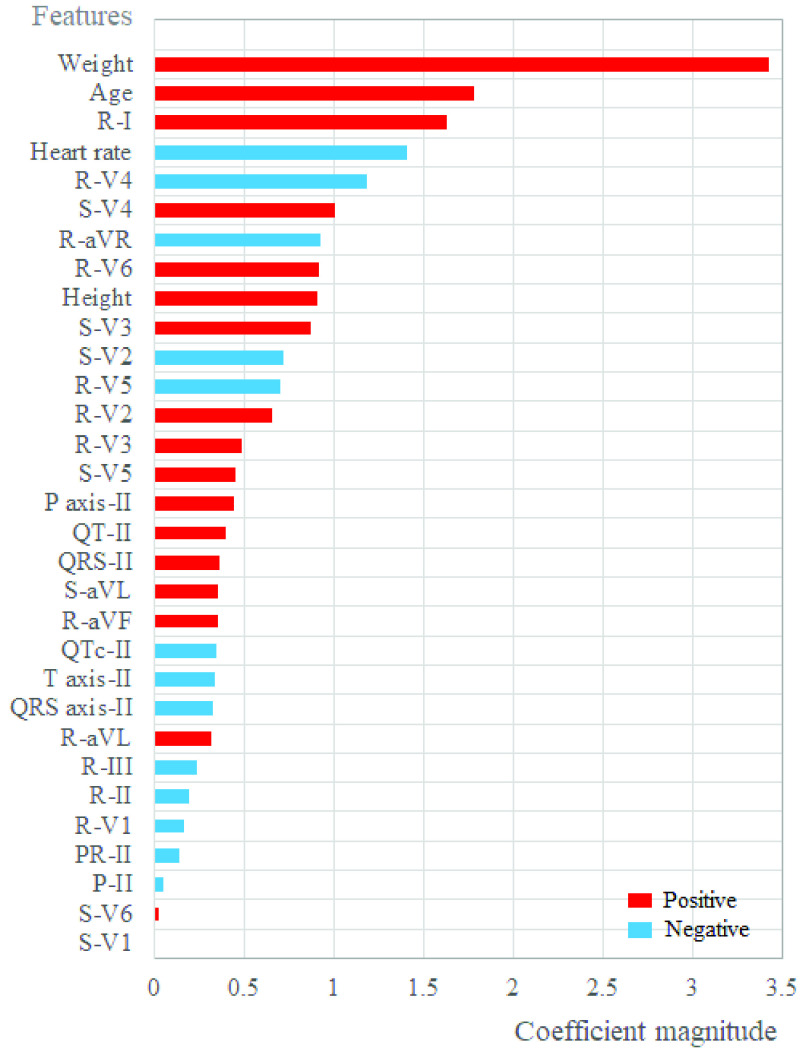
Feature importance of the 31 input parameters.

Fig. 8 exhibits the feature importance in the descending priority with regard to the overall 31 input features. We find that body weight and age are the two most important predictors of echocardiographic RVH in our SVM model. The other important features of RVH with the coefficient magnitude ≥1 include heart rate, the R amplitudes in limb Lead I and chest Lead V4, and the S amplitude in chest Lead V4.

## V. Conclusion

This paper uses machine learning method to train physiological parameters and ECG features in relation to the presence of RVH. We develop a clinically effective ECG system with simple physiological parameters by utilizing the SVM technique to screen RVH in a large sample of young adults. Compared with the traditional ECG criteria including the Sokolow-Lyon voltage and the Myers *et al.* voltage criteria for RVH, the proposed SVM-based technique provides superior performances with regard to sensitivity, precision, F_1_ score, and the AUCs of ROC curves and PR curves. Furthermore, although the proposed model of our ECG-based system is merely trained upon the young males, the SVM-based method can be tested properly for the young females as well. For future work, this proposed ECG-based system with simple physiological parameter inputs will be trained and tested specifically for young females to further clarify the validity and the consistency.
